# Notes on Preparations for the Sick

**Published:** 1910-09

**Authors:** 


### Notes on Preparations for the Sick.

**Tuberculins.**—BURROUGHS, WELLCOME & CO., London.—The signal success which has attended the use of vaccines in staphylococcic and other diseases has had a notable effect in reviving interest and confidence in the specific vaccine treatment of tubercle infections. At first tuberculin as a diagnostic and in certain instances as a curative agent was hailed with enthusiasm by the medical profession, only to be followed soon after by a strong reaction against it, until it was almost entirely given up as a therapeutic agent, though always holding its place as a means of diagnosing tubercular disease. It was left to Koch to obtain a new form of tuberculin, which contained all the curative and immunising substances of the bacilli in a form suitable for inoculation and absorption. Its injection did not produce the dangerous febrile reaction of Old Tuberculin, nor any other ill-effects. New Tuberculin is a slightly opalescent fluid, consisting of an emulsion or suspension of extremely minute particles of bacterial cell-substance. It is readily absorbable, does not cause abscess formation, and is used in the treatment of tubercular affections. The dosage is indicated in terms of weight of tubercle bacilli theoretically represented.

Tubercle Vaccine Bacillary, " Wellcome," is similar to Koch's Bacillary Emulsion, and is prepared in both human and bovine strains, each being issued in two dilutions.

New Tuberculin (W.), human or bovine, is a tubercle " endotoxin " in the sense in which that term has been used by the late Dr. Macfadyen and others. It is intended for use along similar lines to T. R. In the process of preparation the immunising constituents of the bacilli are retained, while the materials which hinder absorption, and thereby tend to bring about local irritation and induration, are removed.

Before dilution Tuberculin (W.) is standardised to contain 2 m. gms. of dry bacillary substances per cc., and the dosage is expressed in terms of the weight of dried bacillary substance actually present. Such indications as are available suggest that weight for weight, Tuberculin (W.) represents approximately five times the dose of Tuberculin (R.).

New Tuberculin (W.) is also issued as a hypodermic tabloid. One tabloid, containing the requisite dose of New Tuberculin, is dissolved in sterile water and injected hypodermically.

**Tuberculin for Von Pirquet's Cutaneous Reaction.**—If the skin of a tuberculous subject be gently scarified as for ordinary vaccination, and tuberculin be applied to the scratched surface, a reaction will occur within twenty-four hours, shown by the appearance of a vivid red papule. Occasionally a large area becomes infiltrated, and a vesicular eruption occurs round the primary area. These signs disappear in a few days. Tuberculin for Von Pirquet's reaction is issued in both human and bovine strains for use according to the nature of the infection suspected.

---

**Nutrient Media—Soloid Brand.**—BURROUGHS, WELLCOME & Co., London.—The preparation of bacteriological culture media on a small scale has always been tedious and unsatisfactory. The time necessary to prepare a few tubes of a given medium is practically as much as would be needed for the preparation of a large bulk, while the loss in filtering small quantities is proportionately very large.

Experiments in the Wellcome Physiological Research Laboratories have shown that many of the ordinary culture media can be dried and kept in this condition for long periods, regaining their original consistency and appearance on being re-dissolved in the requisite amount of water.

Chiefly to meet the needs of the physician or bacteriologist who requires to use a few culture tubes only and at uncertain intervals, the "Soloid" nutrient media have been introduced. These products are dry and portable, will keep indefinitely under proper conditions, and render it possible to prepare a tube or plate of the required medium within an hour without laboratory or apparatus. They are on this account specially suitable for physicians who have not the resources of a laboratory at their disposal. Medical officers of health in rural districts will also find them useful, and for medical expeditions, camps in the tropics, etc., they will meet a decided want.

**"Soloid" Bile-Salt Agar-Agar (MacConkey).**—A very useful solid medium for the isolation of intestinal bacteria, such as B. coli, typhosus and dysenteriæ, from water, milk, fæces, urine, etc. The medium contains bile-salt, peptone, lactose and neutral red. The bile-salts inhibit the growth of most bacteria other than those of intestinal origin. The B. coli colonies are red, but the colonies of B. typhosus and B. dysenteriæ are colourless. The temperature of incubation recommended by MacConkey is 42° C.

**"Soloid" Bile-Salt (MacConkey)** is used for the same purpose as the foregoing when a liquid medium is required.

**"Soloid" Nutrient Broth** provides a fluid medium which may be incubated at any temperature for the cultivation of bacteria. All the common organisms, both pathogenic and non-pathogenic, grow well in this medium.

**"Soloid" Nutrient Gelatin** provides a transparent solid medium when it is desired to determine whether or not the organisms under investigation have any peptonising effect, which is shown by liquefaction of the gelatin.

**"Soloid" (Nutrient) Agar-Agar.**—Prepared by adding agar-agar to peptone beef-broth.

---

**Influenza Vaccine—Wellcome Brand.**—This vaccine is for use in cases of influenza in which the presence of bacillus influenzæ has been demonstrated. An initial dose may be given of 10 million organisms, to be increased according to the indications of the case.

Influenza Vaccine is issued in hermetically-sealed phials as follows :—

(A) 1 c.c. containing 10 million Bacillus Influenzæ.
(B) 1 c.c. ,, 50 million ,, ,,

**Coryza Vaccine—Wellcome Brand.**—Coryza, or catarrh, may be caused by a variety of micro-organisms occurring singly or combined. Amongst those most frequently present are the bacillus septus (bacillus coryza segmentosus) and the micrococcus catarrhalis. Three Coryza Vaccines are issued for use according to the nature of the infection. The initial dose is 100 million organisms of one or both varieties.

Coryza Vaccine is issued in hermetically-sealed phials as follows:—

No. 1. 1 c.c. containing 100 million bacillus septus.
No. 2. 1 c.c. " 100 million micrococcus catarrhalis.
No. 3. 1 c.c. " 100 million bacillus septus and 100 million M. catarrhalis.

---

**Lodal—Tabloids, gr. i.**—Burroughs, Wellcome & Co., London.—" Lodal " is prepared by the oxidation of laudanosine (an alkaloid occurring in opium) in a manner analogous to the preparation of cotarnine from narcotine.

The physiological action of " Lodal " resembles that of cotarnine, in producing tonic contraction in the pregnant and non-pregnant uterus. It differs, however, in that " Lodal " exercises more effect on the heart, slowing and strengthening the beat, and producing a rise in blood-pressure in which vaso-constriction is a definite factor. It has much the same effect on the higher centres, but its action in this respect is more powerful than that of cotarnine. Clinically it has been used with good effect in cases of uterine hemorrhage.

---

**The Tuberculins and their Uses.**—Allen & Hanburys Ltd., London.—To diagnose the presence of tuberculosis by the production of a specific reaction, the " old " Tuberculin of Koch is employed, usually by one of four methods:—

1. By the hypodermic injection of graduated doses.
2. By scarifying the skin and applying a solution of definite strength. (Von Pirquet.)
3. By applying an ointment of Tuberculin over an area of unbroken skin by inunction. (Moro.)
4. By the application of a specially prepared solution to the conjunctiva. (Calmette.)

Every variety and dose is prepared in sterilised " azoules," ready for hypodermic injection, for use either as a diagnostic or curative agent. The pamphlet on the Tuberculins is now in its fifth edition, and much valuable information therein is likely to be of good service.

---

**The " Macnair " Automatic Water Steriliser.**—Allen & Hanburys Ltd., London.—The " Macnair " Automatic Water Steriliser is a simple contrivance, which consists of two cisterns, fitted one on top of the other. The lower is a boiler, practically steam-tight; the upper a locked reservoir. These are connected by a pipe up the centre. The capacity of the reservoir is sufficient for a day's supply, and no water can enter it except through the connecting tube. The boiler is filled with water through a small opening, which is closed by a screw nut. The apparatus is then completely shut and the boiler becomes practically steam-tight. It is then placed on the fire, and when the water boils the steam generated forces the sterilised water up into the reservoir, from whence, after cooling, it can be drawn off by the tap.

---

**Sulphaqua.**—THE SELTZOGENE PATENT CHARGES CO., St. Helens, England.—Sulphaqua is prepared in the form of packets or charges for use in the domestic bath, and a smaller packet is prepared for use in the toilet basin, to make a bath for the face, feet or hands only. The Sulphaqua charges enable the patient to undergo in his own home with equally beneficial results a course of treatment similar to that offered at the Natural Sulphur Spas, without the cost of money attendant on reaching and residing at such locations, and also without the disturbance of business or social engagements.

Sulphaqua with hot water yields a copious and extremely finely divided precipitate of nascent sulphur. Sulphaqua does not blacken the paint of domestic baths.

The baths prepared with this preparation are soothing and non-irritating, they are inoffensive yet efficient, and can be prescribed for the most fastidious patient. They in short offer the advantage of having Harrogate or Strathpeffer brought within one's own home.

A Sulphaqua bath is now a recognised treatment for various skin diseases, rheumatism, gout, sciatica, lumbago, rheumatoid arthritis, neurasthenic conditions in arthritic subjects, etc., and as a valuable addition to the inunction treatment of syphilis with mercury, also in all cases where a course of the natural sulphur baths is indicated.

**Sulphaqua Medicated Soap.**—In disorders of the sebaceous glands, such as seborrhœa or dandruff, comedones or blackheads, hyperidrosis or excessive sweating of the hands or feet, bromidrosis, and for persons subject to eczematous and other skin troubles, the soap will be found of the greatest utility.

---

**"Sanitas-Sypol."**—THE "SANITAS" CO. LTD., Limehouse, London, E., has lately directed attention and experiments to the production of an antiseptic to meet all the requirements of surgical practice.

"Sanitas-Sypol" meets all these, as shown below:—

1. It contains over 50 per cent. of active principles, and is guaranteed to have four times the germicidal efficiency of pure phenol.

2. Diluted with soft or distilled water, it gives a perfectly bright and transparent solution, through which instruments can be discerned as easily as through water.
3. It is non-caustic.
4. It can be diluted 1 in 160 to 1 in 200, and thus diluted is free from slipperiness to the hands and instruments.
5. It has no corrosive or rusting action on metal surgical instruments, or on the hands of the operator.

The "Sanitas" Co. Ltd. are also sole manufacturers of **"Sanitas-Bactox (saponaceous) Disinfectant Fluid"** and **"Sanitas-Okol (emulsion) Disinfectant Fluid."** (Rideal-Walker, co-eff. 20, see *Lancet*, November 27th, 1909, p. 1614.)

---

**Cascara-Agar Jelly.**—BURNETT & TURNER, Clifton.—Many references have been made in recent medical literature to the use of Agar-Agar in habitual constipation. Since Agar-Agar is for the most part excreted unchanged in the fæces on account of its powers to absorb and retain moisture, and to resist the action of intestinal bacteria and enzymes, it prevents the formation of scybalous masses and imparts a soft consistency to the rectal contents. Casc-Agar Jelly is a pleasant and palatable form, in which the jelly is combined with a small proportion of Cascara Sagrada. It will be found that by the use of this preparation it is possible to transform the hard and dry fæces into a softer consistence, so that the intestinal contents are enabled to develop their own agency necessary to produce a healthy, normal evacuation.

It is recommended that one or two teaspoonfuls should be taken night and morning for several days in succession.

---

**Digestin.**—BURGOYNE, BURBIDGES & CO., London; YENJO SHOTEN, Tokyo.—Digestin is a purely digestive ferment procured from the Okazaki fungus, and is prepared in the Pharmaceutical Laboratory at Somei, Sugamo, Japan, some little distance from the city of Tokyo. It possesses mycelium of a whitish appearance, and is obtained from the above-named fungus, which belongs to the order of Aspergillus.

Digestin dissolves in one hour one hundred times its own weight of purified gelatine. The solution gives a marked biuret reaction. It liquefies one hundred times its own weight of starch into dextrine and maltose within eight minutes, and will convert over forty parts of starch into sugar (maltose) in one hour.

The dose for an adult is about 0.3 grammes after each meal.

---

**Potash-Sulphur Water.**—HOT SPRINGS, Ark.; INGRAM AND ROYLE, London.—An alkaline, sodic, sulphated, carbonated and bicarbonated water—a very pure water, free from organic contamination; it flows from the south side of the Ozark Spurs of Arkansas, U.S.A. The uses of sulphur are described as follows:—

1. Sulphur plays an important part in the normal physiological processes of the body, hence it follows that sulphur is an essential to the health of all organs and tissues, and an important element to nutrition.
2. Sulphur is one of the best disinfectants, and as sulphuretted hydrogen is absorbed from the intestinal mucous membrane and carried by the blood to the lungs, where it escapes to the bronchial tubes, it follows that in this passage the sulphur acts as a disinfectant to the lungs and bronchial tubes, which explains its value in consumption and bronchitis.
3. Sulphur stimulates the bile-producing function of the liver, hence its value in torpid liver, constipation and piles.
4. Sulphur has a specific action on the skin, and is one of our most potent remedies in acne, psoriasis, eczema and syphilis.

Make sure your water is good, then drink. If you cannot go to the source, drink at home.

